# Moving Toward a National Policy on Palliative and End of Life Care

**DOI:** 10.4103/0973-1075.76242

**Published:** 2011-01

**Authors:** Stanley C Macaden

**Affiliations:** Coordinator for Palliative Care, Christian Medical Association of India (CMAI), New Delhi, India

**Keywords:** End of life care, Palliative, National policy

## Abstract

Indian Palliative Care has developed over the past 17 years but it has also developed disabilities due to lack of a National Policy and hence has compromised its effectiveness. It is true that we have come a long way but we still have many miles to go and we will get there only if we have a proper road map and sign posts. This article attempts to suggest some specific measures in establishing such a National Policy

## INTRODUCTION

Palliative and end of life care have become an important aspect of health care worldwide.[1] Though this was pioneered and developed by Dame Cicely Saunders in the United Kingdom since 1967, it was taken up by WHO only in the mid-1980s and given the deserved importance and attention. WHO recommended that to be effective there must be intervention through three important areas such as a policy, education, and drug availabilty, policy being the base.[2] Good policies lay the groundwork for an effective health care system and society. They facilitate the implementation of palliative care programs aimed at providing care for all people in need of these services, and they ensure equitable access to affordable medications and therapies. The lack of good policies can lead to unnecessary suffering and costs for patients, families, and society.[3] Many nations committed to provide this service and have succeded by making policies and guidelines at a national level. The Indian Association of Palliative Care is now 17 years old and though the Kerala State Government has declared a policy on Pain and Palliative Care[4] there is no Policy at a national level on Palliative and End of Life Care. Palliative care is mentioned in the National Cancer Control Programme but specific guidelines are lacking. Palliative Care is also needed in other incurable conditions such as AIDS and end stage chronic medical diseases but these remain neglected.

Before formulating a policy on palliative and end of life care, it is important to understand what these terms are. The WHO definition of Palliative Care is clear and well understood. It is also agreed that palliative care begins at the time of diagnosis of an incurable disease. However, the term “End of Life Care” is not very clear. According to the Gold Standards Frame Work, UK, it begins around a year or so when deterioration has started and the answer to the surprise question-would you be surprised if this patient were to die in the next 12 months? Is negative.[5] However, for a lay person and for many health professionals’ “End of Life Care” usually means care in the last hours and days of life. This terminal phase or phase of actively dying is now addressed by an Integrated Care Pathway (ICP) for the dying such as the Liverpool Care Pathway (LCP) for the dying.[6]

**Figure 1:**
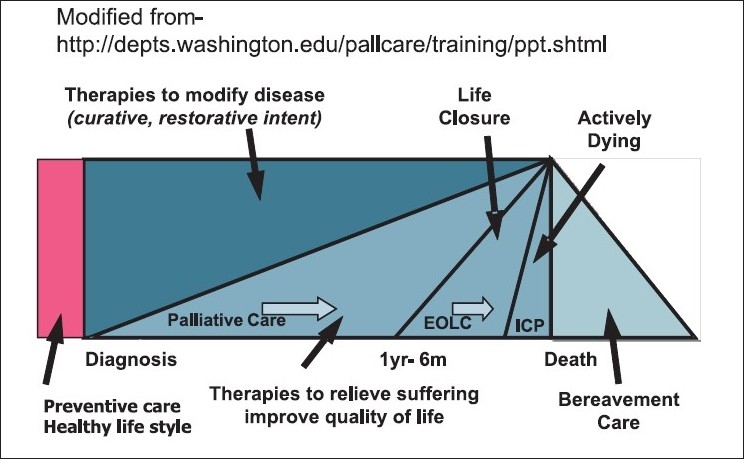
The contimuum of palliative care

It is important that the policy addresses these differences and takes a comprehensive approach to provide the continuum of palliative care that is needed. This is illustrated in the following diagram [Figure 1].

## Steps to move forward

The Indian Association of Palliative Care has already done some splendid work on different aspects of a required Palliative and End of Life Care Policy. What remains now is to put all elements together and complete them and finalize the policy.

1. IAPC has already worked out the “Standards” for palliative care.
2. Various aspects of drug (Morphine) availability have been addressed and guidelines formulated.[7]
3. Education of health professionals in palliative care is now more organized. Inclusion of palliative care in medical, nursing, and allied health care curriculums needs to be achieved.
4. Family involvement is a great strength in our Indian situation and we have a position statement in our Indian Journal of Palliative Care to support care given at home by family.[8]
5. The Neighborhood Network in Palliative Care (NNPC) is a great model to harness the strength of the community and lessons learnt from this can be incorporated.[9]
6. The Indian Society of Critical Care Medicine (ISCCM) has given their position statement on stopping futile treatment in our Intensive Care Units and handing over for palliative care.[10]
7. Piloting of an adapted LCP as the Indian Integrated Care Pathway (IICP) for the dying has taken place in a few centers and experience of other centers is being collated. This needs to be finalized.
8. Keeping with the theme of IAPC Conference, Lucknow, 2011 of “Networking in Palliative care,” this would be a good time for IAPC to network and join hands with the following:
  - With the Indian Society of Critical Care Medicine (ISCCM) to guide the situation in our Indian Intensive Care Units through their position statement and also use this to make inroads into areas of non-cancer palliative care.
  - With the National AIDS Control Organisation (NACO) to make palliative care an integral part of care for people living with HIV/AIDS.
  - With the Indian Society of Nephrology to make palliative care an option for end-stage kidney disease patients who cannot have dialysis.
  - With the Law Commission of India to expedite ACTs to support palliative care decisions and interventions for the terminally ill.
9. IAPC can spearhead a joint consultation of all the above and formulate a National Policy for Palliative and End of Life Care. We are also privileged to have a WHO Collaborating Centre at the Institute of Palliative Medicine in Calicut to facilitate such a joint consultation.
10. This National Policy could then be given to the Government for adoption and to the National Accreditation Board for Hospitals and Health Care (NABH) for implementation in all hospitals in India.
11. This could also become an integral part of the National Cancer Control Program under the National Health Policy.

## CONCLUSION

Formulating a National Policy on palliative and end of life care is long overdue. The IAPC can easily achieve this through a task force by putting together all that has been already achieved and completing some unfinished areas. After this, IAPC can network and join hands with others through a joint consultation to formulate a National Policy. The Government on adoption of the policy can depend on NABH for its implementation in all hospitals and health care set ups in India. Existing programs can then be audited and new programs can be guided so that both aspects of quality and coverage are reasonably and effectively addressed. It will also make Palliative Care available for all those who need it irrespective of their disease. Yes, we must be “Salt” and “Light”[11] and make the difference for the suffering millions in our country for it is their human right to have palliative care and relief of pain.
